# Explainable machine learning for diagnosing severe *Mycoplasma pneumoniae* pneumonia in children: model development and internal validation

**DOI:** 10.3389/fped.2026.1895967

**Published:** 2026-08-14

**Authors:** Yu Zhang, Guihua Chen, Hui Wang, Xinyi Li

**Affiliations:** Department of Pediatrics, The Third People’s Hospital of Chengdu, Chengdu, Sichuan, China

**Keywords:** children, diagnostic model, explainable artificial intelligence, machine learning, mycoplasma pneumoniae pneumonia, random forest, severe pneumonia, XGBoost

## Abstract

**Background:**

*Mycoplasma pneumoniae* pneumonia (MPP) is common in children, but severe MPP (SMPP) may progress rapidly and is difficult to distinguish from non-severe disease because clinical, laboratory, and radiographic findings overlap. We aimed to develop and internally validate machine-learning models for adjunctive SMPP risk stratification using routine data while minimizing circular reasoning.

**Methods:**

We retrospectively included consecutive children with MPP admitted from January 1, 2015, to January 1, 2026. Demographic, clinical, laboratory, and radiographic variables were collected. Variables overlapping with the severity definition, severity-proximal biomarkers, and model-derived leakage variables were excluded before modeling. Eight supervised algorithms were developed in a training cohort and evaluated in an internal test cohort using the area under the receiver operating characteristic curve (AUC), accuracy, sensitivity, specificity, F1-score, calibration, Brier score, and decision curve analysis.

**Results:**

The cohort included 1,046 children, of whom 205 had SMPP; 784 were assigned to the training set and 262 to the internal test set, including 51 SMPP cases. In the strict non-overlap test set, the support vector machine achieved the highest AUC of 0.947 [95% confidence interval (CI), 0.907–0.980], with accuracy of 0.924, sensitivity of 0.784, specificity of 0.957, F1-score of 0.800, and Brier score of 0.058. Random forest achieved an AUC of 0.926 (95% CI, 0.878–0.964), accuracy of 0.897, sensitivity of 0.824, specificity of 0.915, and Brier score of 0.094. It was retained for calibration, decision-curve, threshold, and feature-importance analyses because of its interpretability and balanced performance. Important predictors included aspartate aminotransferase, alanine aminotransferase, albumin, cough duration, blood urea nitrogen, white blood cell count, platelet count, age, wheezing, and lung rales.

**Conclusion:**

After exclusion of leakage variables and severity-definition-overlapping predictors, machine-learning models maintained good internal performance for classifying SMPP in children with MPP. They should be considered adjunctive risk-stratification tools rather than standalone early diagnostic tools. Multicenter external validation and prospective workflow evaluation are required before clinical implementation.

## Introduction

*Mycoplasma pneumoniae* pneumonia (MPP) is a major cause of community-acquired pneumonia in children. Although most cases are self-limited, a subset progresses to severe MPP (SMPP), characterized by persistent fever, hypoxemia, extensive pulmonary inflammation, pleural effusion, atelectasis, necrotizing changes, or extrapulmonary involvement (1–3). Early manifestations often overlap with non-severe disease, which can delay risk recognition and escalation of care.

Clinical assessment of SMPP integrates symptoms, inflammatory markers, and imaging findings. Prolonged fever, elevated C-reactive protein (CRP), lactate dehydrogenase (LDH), neutrophil predominance, serum amyloid A (SAA), and radiographic consolidation have been associated with severe disease (4, 5). However, these indicators are interrelated and may vary with illness duration, prior treatment, sampling time, and radiological interpretation, limiting the reliability of single-variable assessment.

Machine-learning methods can integrate multidimensional clinical data and capture nonlinear relationships. Logistic regression, random forest, gradient-boosting algorithms, support vector machine, and k-nearest neighbors have been applied to pediatric infectious-disease and pneumonia prediction tasks (6–8). Nevertheless, clinically credible models require more than discrimination measured by the area under the receiver operating characteristic curve (AUC); calibration, clinical net benefit, transparent feature processing, and prevention of data leakage are also essential. Inclusion of outcome-derived variables, post-model probabilities, or predictors unavailable at the intended decision point can produce overly optimistic performance.

Previous machine-learning studies of pediatric SMPP have reported promising performance, but many were limited by small samples, single-algorithm designs, incomplete calibration or clinical-utility assessment, and limited interpretability. Few studies have compared multiple commonly used algorithms within one cohort while jointly evaluating discrimination, calibration, Brier score, decision curve analysis, and the risk of circular reasoning. A more rigorous comparison is therefore needed to determine whether routinely available, non-definitional variables can support clinically interpretable risk stratification.

Accordingly, this study aimed to develop and internally validate supervised machine-learning models for pediatric SMPP using routinely available clinical information. We excluded model-derived leakage variables and conducted a strict non-overlap analysis that removed severity-defining clinical/radiological variables and severity-proximal biomarkers from the feature set. The objective was to provide an interpretable and clinically cautious adjunctive risk-stratification framework rather than a standalone replacement for clinical judgment.

## Materials and methods

### Study design and setting

This retrospective model-development and internal-validation study reviewed consecutive eligible children admitted between January 1, 2015, and January 1, 2026. The study was designed to develop and compare machine-learning models for identifying SMPP among children with confirmed MPP. Reporting followed the general principles of the Transparent Reporting of a multivariable prediction model for Individual Prognosis Or Diagnosis (TRIPOD) statement, with emphasis on participant selection, candidate predictors, model development, model performance, calibration, clinical utility, and internal validation (9).

### Participants

Children were eligible if they were aged from 28 days to 14 years, had a diagnosis of MPP during the study period, and had available clinical records, laboratory test results, and chest imaging information from the same disease episode. MPP was diagnosed according to clinical manifestations compatible with respiratory infection together with laboratory evidence of *M. pneumoniae* infection, including positive *M. pneumoniae* deoxyribonucleic acid (DNA) testing and/or a fourfold or greater increase in *M. pneumoniae* antibody titer between acute and convalescent samples when available. Children were excluded if key clinical or laboratory data were seriously incomplete; if they had severe chronic underlying diseases that could substantially affect the clinical course of pneumonia, such as congenital heart disease, immunodeficiency, or chronic respiratory disease; or if tuberculosis coinfection was documented.

### Definition of SMPP

The presence of SMPP was the outcome of interest. Instead of machine-learning outputs, the final clinician-confirmed diagnosis and pediatric guideline-based criteria documented in the medical record were used to classify severity. SMPP was defined using explicit chart-documented evidence of severe illness, including persistent high fever, clinically significant respiratory distress requiring escalation of monitoring, hypoxemia requiring oxygen assessment or support, extensive pulmonary involvement or complications on chest imaging, extrapulmonary involvement, poor mental status, or escalation of inpatient management. Laboratory biomarkers such as CRP, LDH, D-dimer, interleukin-6 (IL-6), and SAA were regarded as supportive markers of inflammatory or tissue-injury burden but were not used as stand-alone criteria to determine the outcome. Children with confirmed MPP were assigned to the non-severe MPP group if they did not meet the clinician-confirmed severity criteria.

### Data collection and candidate predictors

Candidate predictors were selected according to clinical relevance and availability in routine pediatric practice. For descriptive baseline analysis, demographic variables, symptoms, oxygenation status, radiological findings, complications, coinfection, and laboratory markers were summarized. For the strict non-overlap modeling analysis, variables that directly overlapped with SMPP severity-defining criteria were excluded from model training, including fever duration, maximum body temperature, dyspnea, peripheral oxygen saturation, radiological severity score, pleural effusion, extrapulmonary complications, and poor mental status. Severity-proximal biomarkers, including neutrophil percentage, CRP, SAA, LDH, D-dimer, and IL-6, were also excluded. Retained predictors included age, sex, non-definitional clinical variables, *M. pneumoniae* confirmation variables, and routine laboratory markers such as white blood cell count, platelet count, alanine aminotransferase (ALT), aspartate aminotransferase (AST), blood urea nitrogen (BUN), and albumin.

### Radiological assessment and severity scoring

Radiological severity was assigned using a prespecified 1–6 ordinal scale based on the extent and complexity of chest imaging abnormalities documented during the same disease episode. A score of 1 indicated no obvious parenchymal lesion or only minimal peribronchial/interstitial changes; 2, focal unilateral patchy infiltrates without lobar consolidation; 3, segmental or lobar consolidation limited to one lobe; 4, multilobar unilateral disease or bilateral patchy infiltrates/consolidations without major complication; 5, extensive bilateral or multilobar consolidation, pleural effusion, atelectasis, or suspected necrotizing change; and 6, diffuse bilateral disease or major radiological complications, such as large pleural effusion/empyema, necrotizing pneumonia, or marked atelectasis requiring urgent imaging reassessment. Higher scores therefore represented more extensive pulmonary involvement.

Radiological variables were extracted from formal chest radiography or chest computed tomography (CT) reports issued during routine care. To standardize retrospective scoring, two investigators independently mapped imaging descriptions to the prespecified scale according to a written extraction rule and without access to model predictions. Disagreements were resolved through discussion and, when necessary, consultation with a senior pediatric clinician or radiologist. The radiological severity score was retained for descriptive baseline characterization but excluded from the strict non-overlap modeling feature set because it overlapped with the clinician-confirmed severity definition.

### Data preprocessing and leakage control

An examination of the dataset was performed before model construction to identify duplicate records, impossible values, severe outliers, variable-format problems, and missing values. Continuous data were screened using distribution plots and clinically plausible ranges, whereas categorical variables were examined for inconsistent coding. Missing values were handled within the training workflow using procedures appropriate to variable type and missingness pattern. To prevent data leakage, variables generated directly from the outcome or from previous model predictions were removed before dataset splitting and model fitting. Specifically, simulated risk scores, model-predicted probabilities, model-predicted labels, and prediction-note variables were excluded from all models. In addition, the revised strict non-overlap analysis excluded clinical/radiological variables and biomarkers that overlapped with the outcome definition to reduce circular reasoning, in accordance with current concerns regarding bias in artificial-intelligence prediction models (10). Detailed variable definitions, coding schemes, missingness, and preprocessing strategies are provided in Supplementary Table S1.

### Dataset splitting and class imbalance handling

Eligible participants were separated by stratified sampling into a training set (75%) and an independent internal test set (25%), while maintaining a similar proportion of SMPP cases in both subsets. To address class imbalance, class-weighted algorithms were used when available. For algorithms that did not naturally handle class imbalance, model tuning emphasized balanced discrimination and clinically meaningful sensitivity rather than overall accuracy alone. All preprocessing steps capable of learning from data distributions were fitted within the training workflow to avoid information leakage from the test set.

### Machine-learning model development

We developed and compared multiple supervised machine-learning algorithms, including logistic regression, random forest, XGBoost, LightGBM, support vector machine, k-nearest neighbors, decision tree, and Gaussian naive Bayes. Logistic regression was included as a transparent baseline model. Tree-based ensemble models were included because of their ability to capture nonlinear relationships and interactions, whereas support vector machine and k-nearest neighbors were included for broader algorithmic comparison. Hyperparameters were selected within the training set using cross-validation. The random forest model used for threshold, calibration, decision-curve, and feature-importance analyses included 500 trees, a maximum depth of six, a minimum leaf size of ten, and class-weight balancing. The machine-learning algorithms and principal hyperparameters are detailed in Supplementary Table S2.

### Internal validation and model comparison

Five-fold cross-validation was applied within the training set. In each fold, the model was trained on four-fifths of the training data and evaluated on the remaining fold. Cross-validation performance was summarized using the mean AUC and variability across folds. After hyperparameter tuning, each model was refitted in the full training set and evaluated in the independent internal test set. Model comparison considered discrimination, classification accuracy, sensitivity, specificity, precision, F1-score, probability calibration, and clinical utility.

### Performance metrics

Discrimination was assessed using the AUC with 95% confidence intervals (CIs). Classification performance at selected probability thresholds was evaluated using accuracy, sensitivity, specificity, positive predictive value, negative predictive value, precision, recall, and F1-score. Confusion matrices showed true-negative, false-positive, false-negative, and true-positive predictions. Calibration was assessed using calibration plots and the Brier score, with lower Brier scores indicating better probabilistic accuracy. Decision curve analysis was used to assess the potential clinical net benefit of the final model across a range of threshold probabilities (11). For practical interpretation, several random forest probability thresholds were examined, including a high-sensitivity outpatient-screening threshold, an emergency-triage threshold, a balanced diagnostic threshold, and a high-specificity warning threshold.

### Model interpretability

To improve clinical interpretability, feature-importance analysis was performed for the strict non-overlap random forest model. Predictor importance was summarized according to the relative contribution of each retained feature to model performance. Because severity-defining clinical/radiological variables and severity-proximal biomarkers were excluded, the importance ranking was interpreted as the contribution of non-overlapping variables rather than as evidence that the model rediscovered the clinical definition of SMPP.

### Statistical analysis

Continuous variables were described as mean ± standard deviation or median with interquartile range depending on distribution, and categorical variables were described as number and percentage. Between-group comparisons were performed using the t test or Mann–Whitney U test for continuous variables and the chi-square test or Fisher exact test for categorical variables, as appropriate. A two-sided P value less than 0.05 was considered statistically significant. Machine-learning analyses were performed using Python with standard scientific-computing and machine-learning libraries, including pandas, NumPy, scikit-learn, XGBoost, LightGBM, and matplotlib. Random seeds were fixed during dataset splitting and model training to improve reproducibility.

## Results

### Study population and baseline characteristics

**Figure 1:**
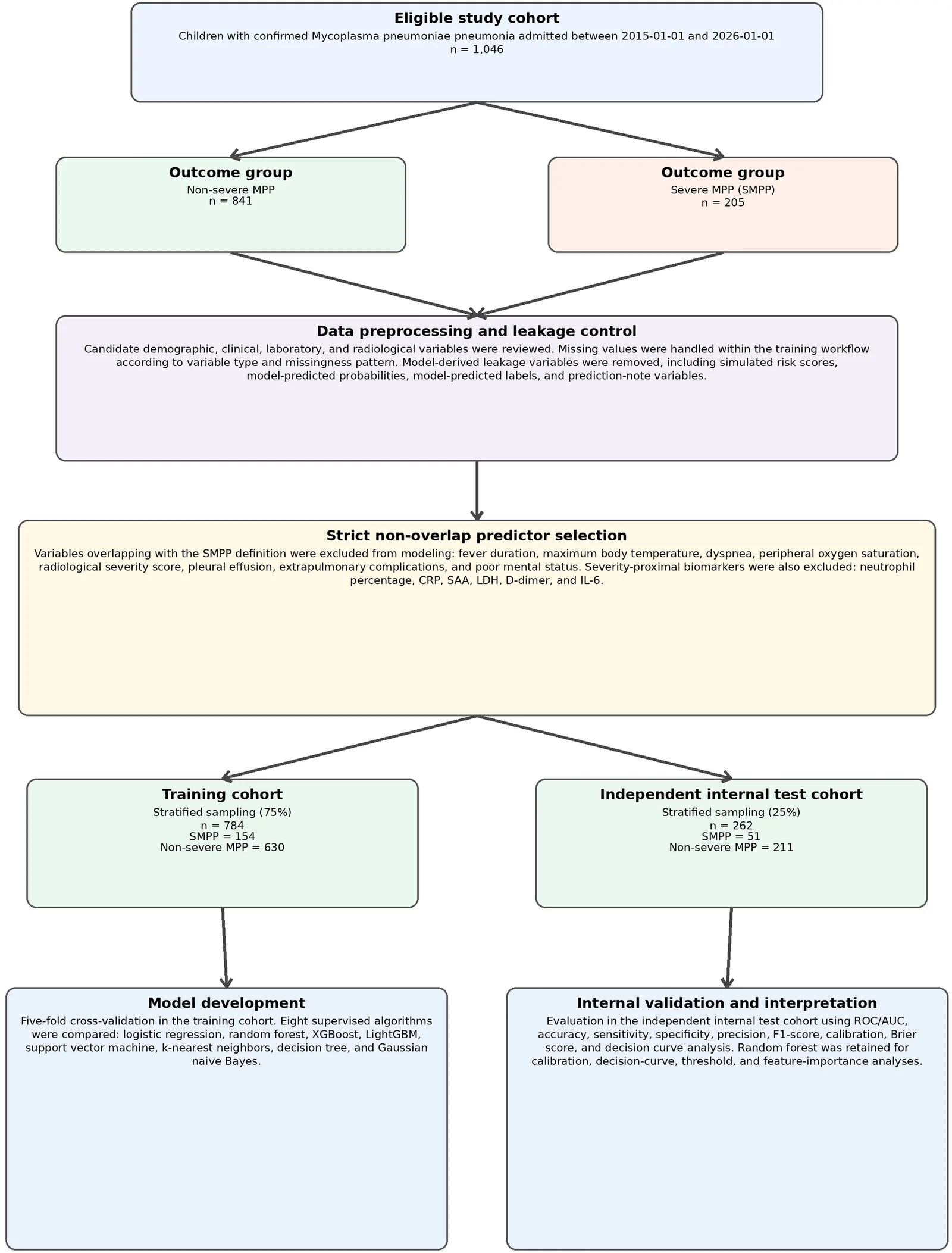
Study workflow for development and internal validation of machine-learning models for classifying severe *Mycoplasma pneumoniae* pneumonia among 1,046 children with confirmed *Mycoplasma pneumoniae* pneumonia. The flowchart summarizes participant inclusion, data preprocessing, exclusion of leakage and severity-definition-overlapping variables, strict non-overlap predictor selection, model development, internal validation, model comparison, calibration, decision curve analysis, and interpretability analysis.

The final cohort comprised 1,046 children with MPP, including 841 with non-severe MPP and 205 with SMPP (19.6%). Stratified sampling assigned 784 children (154 SMPP and 630 non-severe MPP) to the training set and 262 children (51 SMPP and 211 non-severe MPP) to the internal test set. Figure 1 summarizes participant inclusion, data preprocessing, leakage-variable exclusion, strict non-overlap predictor selection, model development, internal validation, model comparison, calibration, clinical-utility assessment, and model interpretation.

Baseline clinical, laboratory, and radiological characteristics are presented in Table 1. Age and sex distribution were similar between the non-severe MPP and SMPP groups. Children with SMPP had longer fever duration (7.5 ± 2.3 vs. 5.2 ± 2.0 days), longer cough duration (8.4 ± 3.1 vs. 6.1 ± 2.8 days), and higher maximum temperature (39.3 ± 0.6 °C vs. 38.6 ± 0.7 °C) than those with non-severe MPP. Respiratory distress-related manifestations were also more frequent in the SMPP group, including wheezing (34.6% vs. 19.4%), dyspnea (28.3% vs. 6.8%), and poor mental status (17.1% vs. 3.9%). Oxygen saturation was lower in the SMPP group (94.9 ± 2.8% vs. 97.4 ± 1.8%), and radiological severity was higher (imaging score, 4.4 ± 1.1 vs. 2.9 ± 1.1). Pleural effusion and extrapulmonary consequences were shown to be more prevalent in children who were diagnosed with SMPP. The SMPP group exhibited significantly higher levels of inflammatory and tissue-injury markers, such as the count of white blood cells, the percentage of neutrophils, the platelet count, C-reactive protein (CRP), serum amyloid A (SAA), lactate dehydrogenase (LDH), D-dimer, and interleukin-6 (IL-6). All of these markers were statistically significant (*P* < 0.001), with the exception of coinfection (*P* = 0.018).

**Table 1:** Baseline characteristics of children with non-severe MPP and SMPP.

Characteristic	Non-severe MPP (*n* = 841)	SMPP (*n* = 205)	P value
Age, months	73.6 ± 38.5	67.9 ± 36.8	0.057
Male sex, *n* (%)	455 (54.1)	117 (57.1)	0.434
Fever duration, days	5.2 ± 2.0	7.5 ± 2.3	<0.001
Cough duration, days	6.1 ± 2.8	8.4 ± 3.1	<0.001
Maximum temperature, °C	38.6 ± 0.7	39.3 ± 0.6	<0.001
Wheezing, *n* (%)	163 (19.4)	71 (34.6)	<0.001
Dyspnea, *n* (%)	57 (6.8)	58 (28.3)	<0.001
Poor mental status, *n* (%)	33 (3.9)	35 (17.1)	<0.001
SpO2, %	97.4 ± 1.8	94.9 ± 2.8	<0.001
Imaging score	2.9 ± 1.1	4.4 ± 1.1	<0.001
Pleural effusion, *n* (%)	38 (4.5)	48 (23.4)	<0.001
Extrapulmonary complications, *n* (%)	29 (3.4)	38 (18.5)	<0.001
Coinfection, *n* (%)	181 (21.5)	60 (29.3)	0.018
WBC, ×10⁹/L	8.1 ± 2.6	10.2 ± 3.4	<0.001
Neutrophil, %	57.8 ± 14.7	68.9 ± 13.1	<0.001
Platelet, ×10⁹/L	287 ± 86	329 ± 102	<0.001
CRP, mg/L	24.5 (12.4–45.7)	66.8 (38.9–112.4)	<0.001
SAA, mg/L	64.2 (29.6–121.3)	151.7 (78.4–264.1)	<0.001
LDH, IU/L	302 ± 94	421 ± 132	<0.001
D-dimer, mg/L	0.82 (0.47–1.42)	1.71 (0.92–3.08)	<0.001
IL-6, pg/mL	13.5 (6.4–29.8)	34.6 (16.8–72.1)	<0.001

The corrected analysis excluded Risk_score_sim, *_pred_probability, *_predicted_label, and Prediction_note columns to avoid label leakage. Values are presented as mean ± SD, median (IQR), or *n* (%), as appropriate.

### Candidate predictors and data preprocessing

As part of the original descriptive predictor set, demographic variables, clinical symptoms, oxygen saturation, radiological severity, complications, coinfection status, and routinely available laboratory indicators were summarized. For the strict non-overlap modeling analysis, the final corrected study excluded not only derived or post-model leakage variables, including simulated risk scores, model-predicted probabilities, model-predicted labels, and prediction notes, but also variables that directly overlapped with the SMPP definition or closely reflected severity. The revised predictor list, inclusion/exclusion status, and rationale are shown in Table 2.

**Table 2:** Candidate predictors and preprocessing strategy after leakage exclusion.

Variable	Type	Coding or unit	Missing rate, %	Preprocessing in corrected analysis
Age	Continuous	Months	0.0	Included
Sex	Binary	Male=1, Female=0	0.0	One-hot/binary coding
Fever duration	Continuous	Days	2.5	Multiple imputation
Cough duration	Continuous	Days	3.3	Multiple imputation
Maximum temperature	Continuous	°C	1.9	Multiple imputation
Wheezing	Binary	Yes=1, No = 0	1.7	Mode imputation
Dyspnea	Binary	Yes=1, No = 0	1.5	Mode imputation
Poor mental status	Binary	Yes=1, No = 0	1.6	Mode imputation
Lung rales	Binary	Yes=1, No = 0	2.4	Mode imputation
SpO2	Continuous	%	4.8	Multiple imputation
Imaging score	Ordinal	1–6 points	3.6	Multiple imputation
Pleural effusion	Binary	Yes=1, No = 0	2.1	Mode imputation
Extrapulmonary complications	Binary	Yes=1, No = 0	1.8	Mode imputation
Coinfection	Binary	Yes=1, No = 0	5.4	Mode imputation
WBC	Continuous	×10⁹/L	3.1	Multiple imputation
Neutrophil percentage	Continuous	%	3.9	Multiple imputation
Platelet	Continuous	×10⁹/L	4.7	Multiple imputation
CRP	Continuous	mg/L	4.2	Multiple imputation
SAA	Continuous	mg/L	7.9	Multiple imputation
LDH	Continuous	IU/L	4.5	Multiple imputation
ALT	Continuous	U/L	5.7	Multiple imputation
AST	Continuous	U/L	5.9	Multiple imputation
BUN	Continuous	mmol/L	6.6	Multiple imputation
Albumin	Continuous	g/L	5.2	Multiple imputation
D-dimer	Continuous	mg/L	8.1	Multiple imputation
IL-6	Continuous	pg/mL	10.8	Multiple imputation
Risk_score_sim and model-prediction columns	Derived/leakage	Not clinical baseline variables	0.0	Excluded from corrected model

The corrected analysis excluded Risk_score_sim, *_pred_probability, *_predicted_label, and Prediction_note columns to avoid label leakage. Values are presented as mean ± SD, median (IQR), or *n* (%), as appropriate.

**Figure 2:**
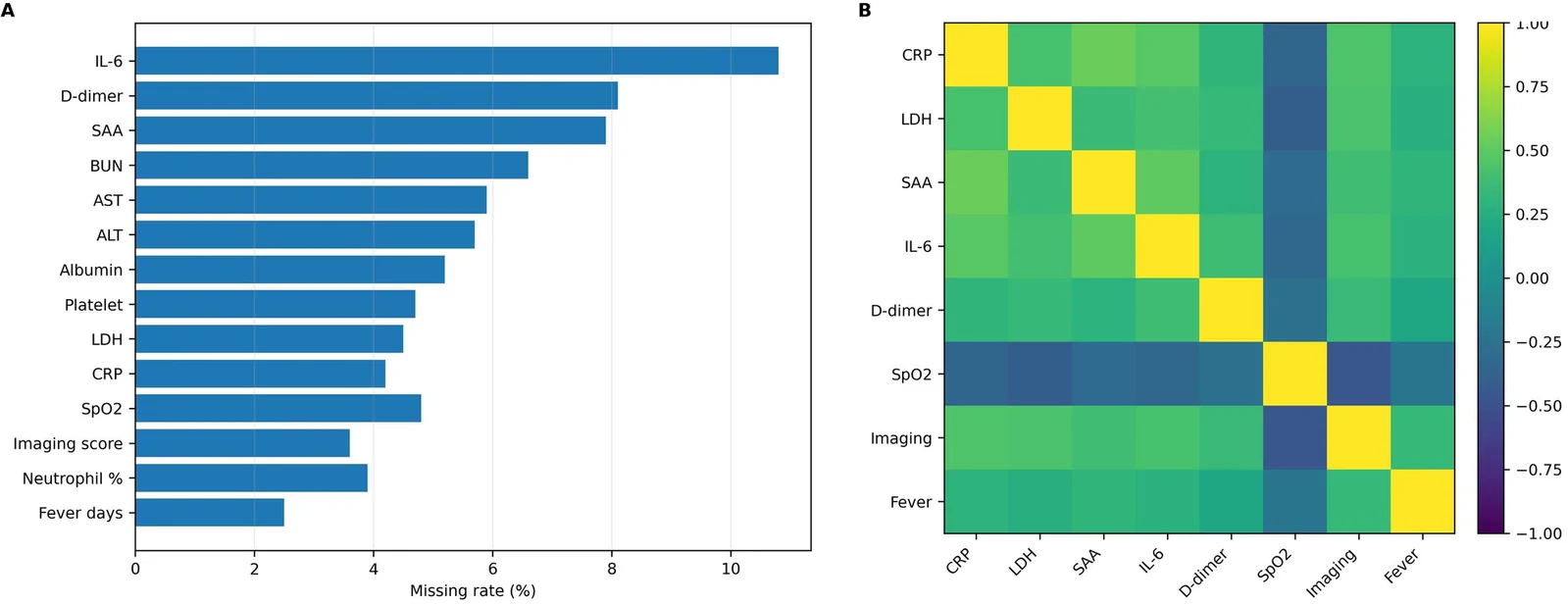
Data-preprocessing diagnostics for candidate predictors in the cohort of children with *Mycoplasma pneumoniae* pneumonia. **(A)** shows the percentage of missing values for each candidate predictor before imputation, and **(B)** shows the Spearman correlation heatmap used to examine pairwise collinearity before application of the strict non-overlap exclusion rules.

The overall missingness of candidate clinical variables was low to moderate. Missing rates ranged from 0.0% for age and sex to 10.8% for IL-6. Continuous variables with missing values were handled by imputation within the training workflow, while binary variables were handled using mode imputation. The missing-value distribution is shown in Figure 2A. A correlation analysis was performed to inspect collinearity among candidate predictors, and no predictor pair required exclusion solely because of excessive collinearity after clinical review (Figure 2B).

### Comparison of machine-learning models

**Figure 3:**
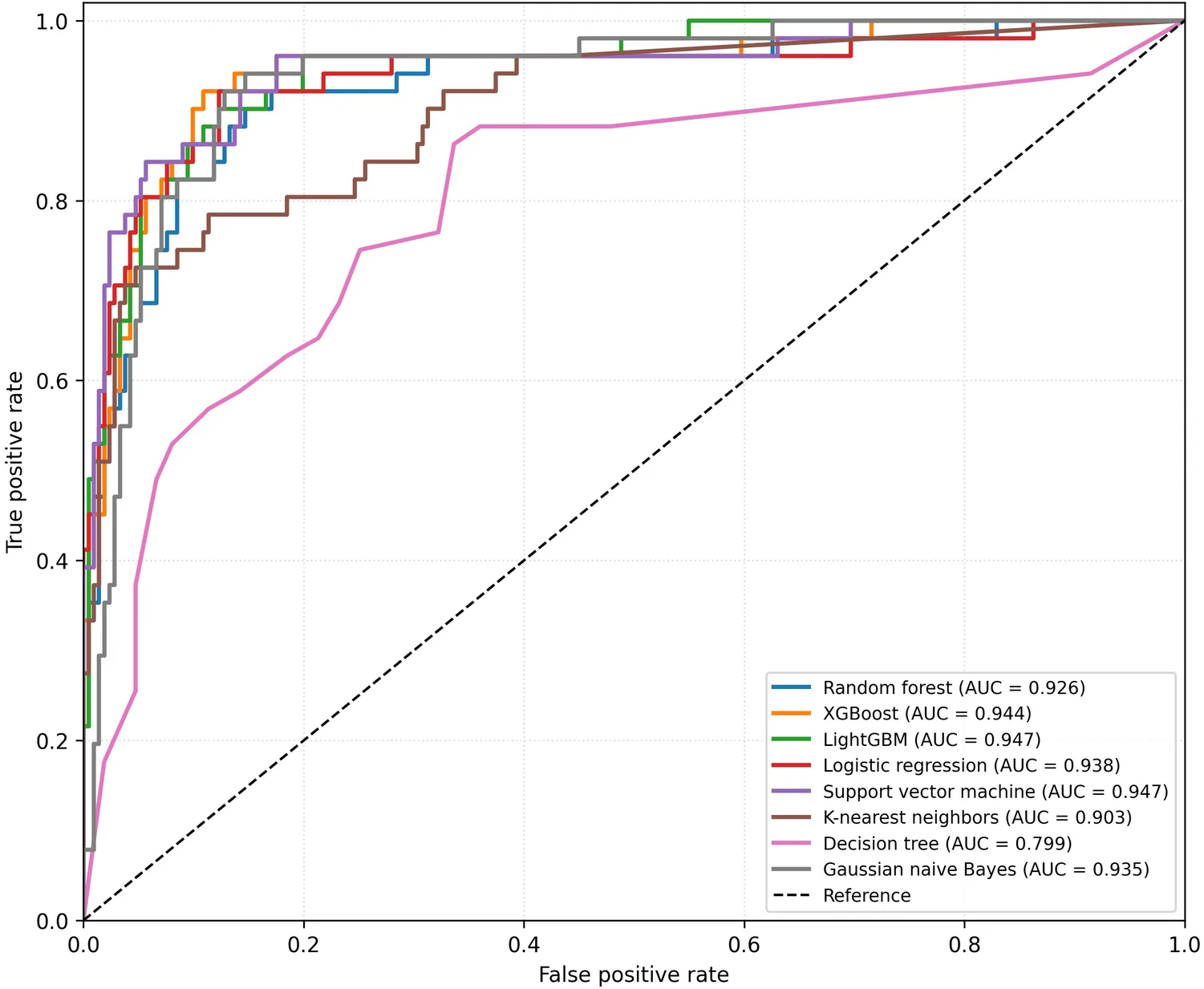
Receiver operating characteristic (ROC) curves of eight strict non-overlap machine-learning models for classifying severe *Mycoplasma pneumoniae* pneumonia in the independent internal test set (*n* = 262; 51 severe cases). The curves compare model discrimination after exclusion of model-derived leakage variables, severity-definition-overlapping predictors, and severity-proximal biomarkers.

The diagnostic performance of different machine-learning algorithms in the strict non-overlap internal test set is shown in Table 3 and Figure 3. Support vector machine achieved the highest AUC of 0.947 (95% CI, 0.907–0.980), with accuracy of 0.924, sensitivity of 0.784, specificity of 0.957, precision of 0.816, F1-score of 0.800, and Brier score of 0.058. LightGBM showed a similar AUC of 0.947 (95% CI, 0.913–0.974), with accuracy of 0.905, sensitivity of 0.843, specificity of 0.919, and F1-score of 0.775. XGBoost achieved an AUC of 0.944 (95% CI, 0.906–0.973). The random forest model achieved an AUC of 0.926 (95% CI, 0.878–0.964), accuracy of 0.897, sensitivity of 0.824, specificity of 0.915, precision of 0.700, F1-score of 0.757, and Brier score of 0.094.

**Table 3:** Machine-learning model performance in the strict non-overlap internal test set.

Model	AUC (95% CI)	Accuracy	Sensitivity	Specificity	Precision	F1-score	Brier score
Random forest	0.926 (0.878–0.964)	0.897	0.824	0.915	0.700	0.757	0.094
XGBoost	0.944 (0.906–0.973)	0.916	0.843	0.934	0.754	0.796	0.073
LightGBM	0.947 (0.913–0.974)	0.905	0.843	0.919	0.717	0.775	0.073
Logistic regression	0.938 (0.889–0.976)	0.878	0.863	0.882	0.638	0.733	0.082
Support vector machine	0.947 (0.907–0.980)	0.924	0.784	0.957	0.816	0.800	0.058
K-nearest neighbors	0.903 (0.857–0.948)	0.851	0.275	0.991	0.875	0.418	0.101
Decision tree	0.799 (0.721–0.869)	0.752	0.686	0.768	0.417	0.519	0.166
Naive Bayes	0.935 (0.898–0.967)	0.901	0.706	0.948	0.766	0.735	0.085

Results are based on the strict Reviewer-2 non-overlap feature set. The uploaded dataset contained 784 training cases and 262 internal test cases, including 51 SMPP cases in the test set. Variables overlapping with severity-defining criteria, severity-proximal biomarkers, and all model-derived leakage variables were excluded before model fitting.

Other models also demonstrated acceptable discrimination. Logistic regression achieved an AUC of 0.938, naive Bayes achieved an AUC of 0.935, k-nearest neighbors achieved an AUC of 0.903, and decision tree achieved an AUC of 0.799. Overall, the receiver operating characteristic curves indicated that several algorithms maintained strong discrimination even after removal of severity-definition-overlapping variables. Because support vector machine showed the highest AUC and Brier score performance, whereas random forest provided clinically interpretable feature importance and balanced threshold behavior, both overall discrimination and interpretability were considered when interpreting the revised model results.

Five-fold cross-validation in the training set supported the stability of the strict non-overlap models. The mean cross-validated AUC was 0.951 ± 0.027 for support vector machine, 0.949 ± 0.029 for logistic regression, 0.944 ± 0.025 for random forest, 0.938 ± 0.022 for LightGBM, 0.936 ± 0.028 for XGBoost, 0.932 ± 0.034 for naive Bayes, 0.908 ± 0.030 for k-nearest neighbors, and 0.864 ± 0.023 for decision tree (Supplementary Table S3). These results were broadly consistent with the independent internal test-set findings. Model-specific confusion matrices and classification performance are provided in Supplementary Table S4.

To address the long study period, an exploratory time-period analysis was performed in the internal test set using the strict non-overlap random forest model. The AUCs were 0.911 for 2015–2019, 0.951 for 2020–2022, and 0.944 for 2023–2026 (Supplementary Table S5). These findings suggest broadly stable internal discrimination across the periods before, during, and after the coronavirus disease 2019 (COVID-19) pandemic, although subgroup estimates should be interpreted cautiously because all data were derived from a single center and subgroup sample sizes were limited.

### Calibration and clinical utility of the final random forest model

**Figure 4:**
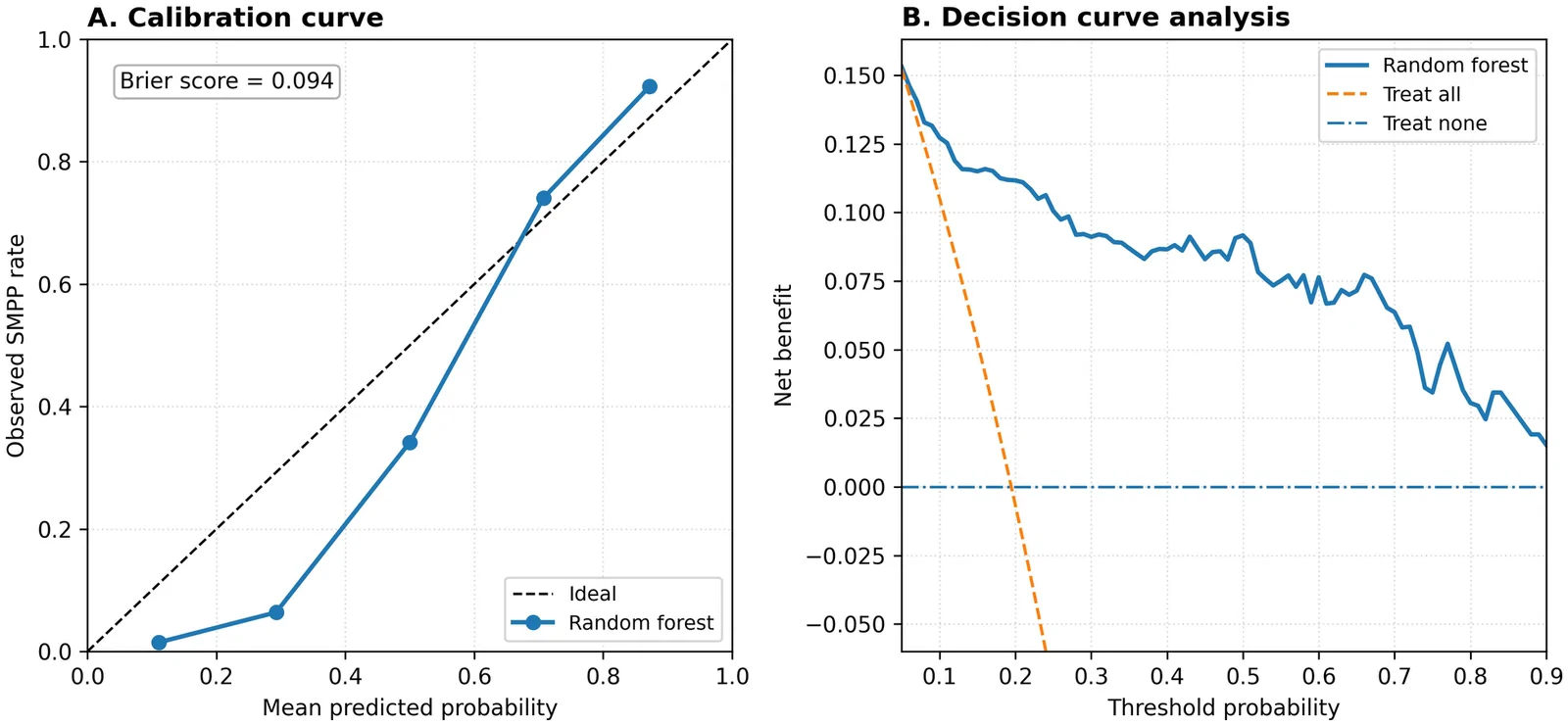
Calibration and decision curve analysis of the strict non-overlap random forest model in the independent internal test set of children with *Mycoplasma pneumoniae* pneumonia. **(A)** compares predicted probabilities with observed severe-disease frequencies, and **(B)** shows clinical net benefit across a range of threshold probabilities relative to treat-all and treat-none strategies.

Figure 4A depicts the calibration curve of the strict non-overlap random forest model. The predicted probabilities were generally consistent with the observed SMPP rates across probability strata, indicating acceptable calibration after exclusion of both leakage variables and severity-definition-overlapping predictors. The Brier score for the random forest model was 0.094, suggesting reasonable probabilistic accuracy.

Decision curve analysis showed that the strict non-overlap random forest model provided positive net benefit across a range of clinically relevant threshold probabilities (Figure 4B). These findings support the potential use of the model as an adjunctive risk-stratification tool after routine clinical assessment, but they should not be interpreted as evidence that the model can independently diagnose SMPP before severity-related clinical information becomes available.

### Threshold analysis of the random forest model

Table 4 summarizes threshold-specific performance of the strict non-overlap random forest model. At a probability threshold of 0.30, the model produced high sensitivity (0.922) and high negative predictive value (0.975), which may be useful when the objective is to reduce missed SMPP cases. At a threshold of 0.40, sensitivity remained high (0.902), while specificity increased to 0.834.

**Table 4:** Threshold-specific performance of the strict non-overlap random forest model.

RF probability threshold	Sensitivity	Specificity	PPV	NPV	Accuracy	TN/FP/FN/TP	Recommended use
0.30	0.922	0.744	0.465	0.975	0.779	157/54/4/47	High-sensitivity screening
0.40	0.902	0.834	0.568	0.972	0.847	176/35/5/46	Emergency triage
0.50	0.824	0.915	0.700	0.955	0.897	193/18/9/42	Balanced diagnostic threshold
0.60	0.627	0.962	0.800	0.914	0.897	203/8/19/32	Confirmatory inpatient assessment
0.70	0.510	0.981	0.867	0.892	0.889	207/4/25/26	High-specificity warning

PPV, positive predictive value; NPV, negative predictive value. The balanced threshold of 0.50 corresponds to the confusion matrix shown in revised Figure 6. Threshold labels are illustrative and should be finalized according to the intended clinical context after external validation.

At the balanced threshold of 0.50, the random forest model achieved sensitivity of 0.824, specificity of 0.915, positive predictive value of 0.700, negative predictive value of 0.955, and accuracy of 0.897. The corresponding confusion matrix included 193 true negatives, 18 false positives, 9 false negatives, and 42 true positives. When the threshold was increased to 0.60, specificity increased to 0.962 and positive predictive value to 0.800, while sensitivity decreased to 0.627. At the high-specificity threshold of 0.70, specificity reached 0.981 and positive predictive value reached 0.867, but sensitivity decreased to 0.510. Threshold selection should therefore depend on the intended clinical context and the relative consequences of false-negative and false-positive classifications.

### Model interpretability and important predictors

**Figure 5:**
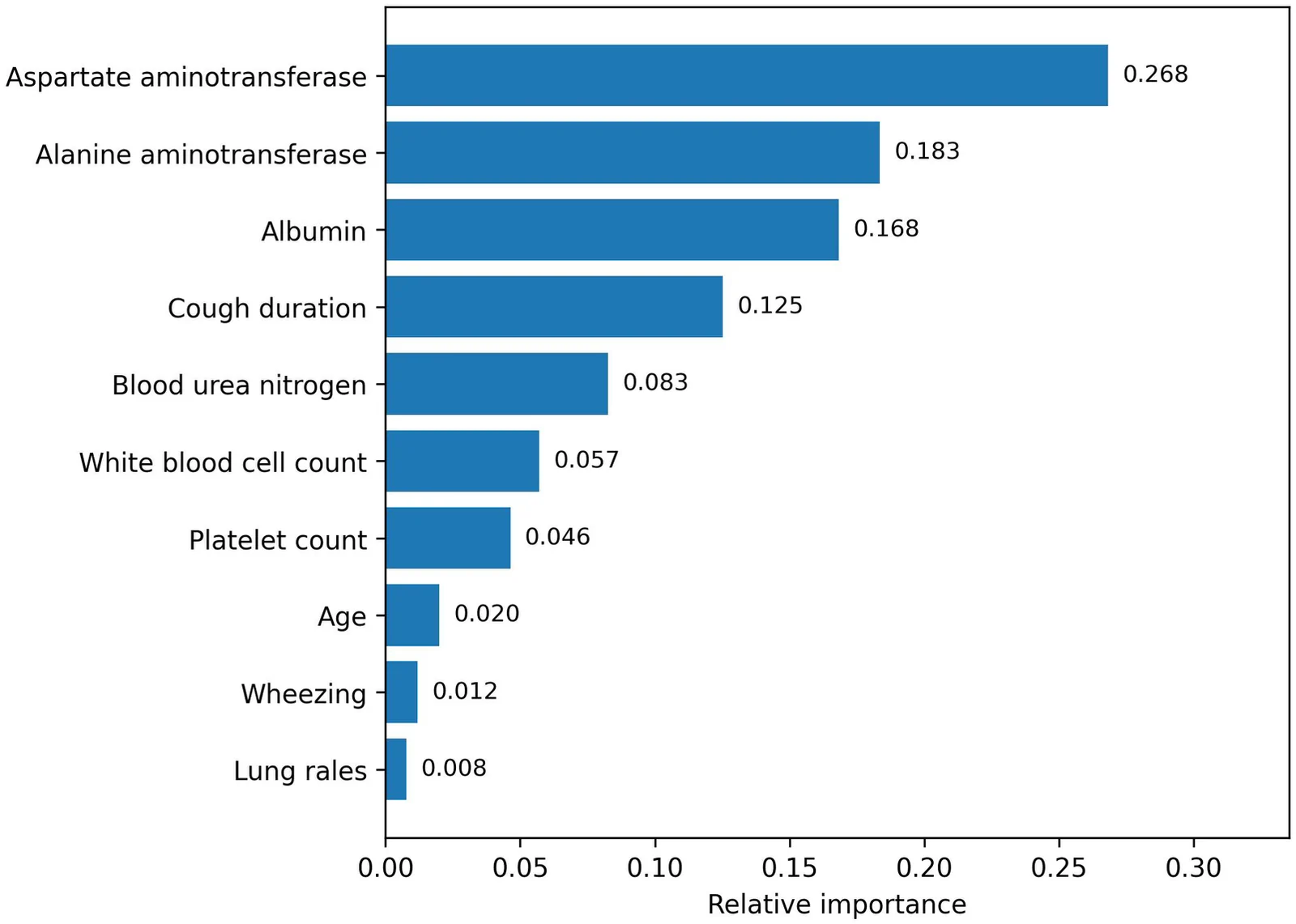
Feature importance of the strict non-overlap random forest model for classifying severe *Mycoplasma pneumoniae* pneumonia. The plot presents the relative contribution of retained non-definitional clinical and laboratory predictors after exclusion of leakage variables, severity-definition-overlapping variables, and severity-proximal biomarkers.

**Figure 6:**
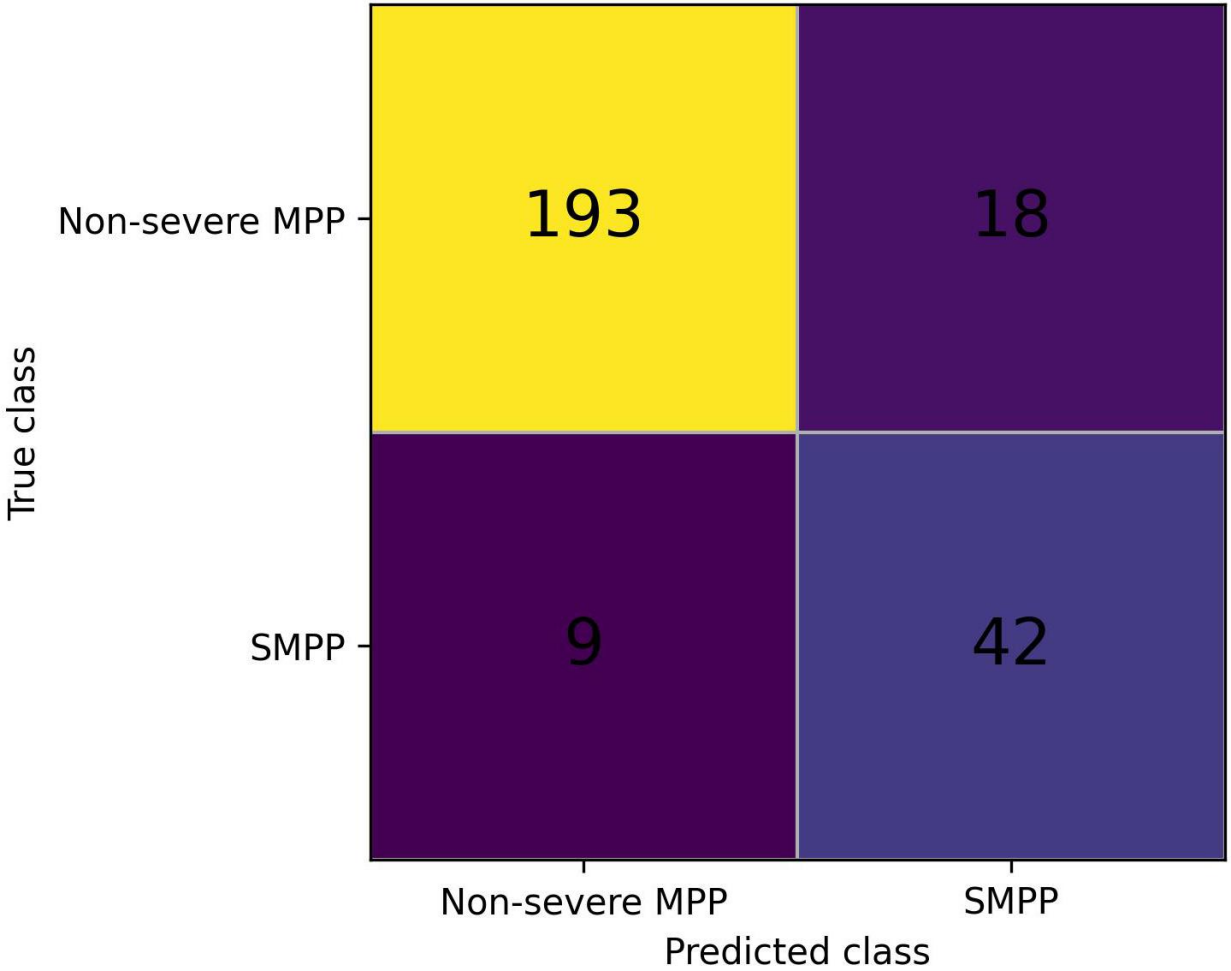
Confusion matrix of the strict non-overlap random forest model in the independent internal test set (*n* = 262) at the balanced probability threshold of 0.50. The matrix reports true non-severe cases, false-positive classifications, false-negative classifications, and true severe *Mycoplasma pneumoniae* pneumonia cases.

Figure 5 displays feature-importance analysis of the strict non-overlap random forest model. The most important retained variable was AST (importance = 0.268), followed by ALT (0.183), albumin (0.168), cough duration (0.125), BUN (0.083), white blood cell count (0.057), platelet count (0.046), age (0.020), wheezing (0.012), lung rales (0.008), chest pain (0.007), and coinfection (0.006). These findings differ from the original model because variables directly overlapping with the SMPP definition, such as radiological severity score, oxygen saturation, dyspnea, persistent fever variables, pleural effusion, extrapulmonary complications, and poor mental status, were excluded from the revised strict model. The corresponding confusion matrix at the balanced probability threshold of 0.50 is presented in Figure 6.

The revised importance ranking should be interpreted cautiously. It does not imply that transaminase levels or albumin alone define SMPP, nor does it replace clinical assessment. Instead, it indicates that, after removal of explicit severity-defining variables, residual information from routine biochemical markers, cough duration, blood counts, and non-definitional clinical findings still contributed to internal classification performance. This revision directly reduces the risk that the model simply reproduced the clinician-confirmed outcome definition.

## Discussion

This retrospective model-development and internal-validation study evaluated machine-learning models for classifying SMPP among children with confirmed MPP while explicitly addressing data leakage and circular reasoning. In the strict non-overlap internal test set, support vector machine achieved the highest AUC of 0.947, whereas the random forest model achieved an AUC of 0.926 with sensitivity of 0.824, specificity of 0.915, and Brier score of 0.094. Importantly, these results were obtained after excluding model-derived leakage variables, severity-definition-overlapping clinical/radiological variables, and severity-proximal biomarkers. The revised findings therefore suggest that non-overlapping routinely available variables can still provide useful internal discrimination, but the model should be regarded as an adjunctive risk-stratification framework rather than a standalone early diagnostic tool. These results are consistent with recent machine-learning and artificial-intelligence studies showing that multidimensional clinical variables and automated imaging features can support classification of severe or refractory M. pneumoniae pneumonia (12–14).

The corrected performance profile requires cautious interpretation. Although an AUC above 0.90 indicates strong internal discrimination, the study remains retrospective and single-center, and the outcome was based on clinician-confirmed severity classification documented in routine care. The threshold analysis is clinically informative because lower thresholds improved sensitivity and negative predictive value, whereas higher thresholds improved specificity and positive predictive value. However, these thresholds are illustrative and should not be directly implemented without external validation, prospective calibration monitoring, and assessment of clinical consequences (15, 16).

### Interpretation of key predictors

In the strict non-overlap random forest model, the most influential retained variables were AST, ALT, albumin, cough duration, BUN, white blood cell count, platelet count, age, wheezing, and lung rales. This revised ranking is substantially different from the original feature-importance profile because predictors that directly formed part of the severity definition were deliberately removed. Therefore, the feature-importance analysis should no longer be interpreted as discovering that hypoxemia, extensive radiological involvement, or respiratory distress predict SMPP. Instead, the revised analysis assesses whether non-definitional variables contain residual information that helps classify clinician-confirmed severity. Previous pediatric studies have similarly linked lactate dehydrogenase, macrolide resistance, treatment response, and broader clinical profiles with severe, necrotizing, or unresponsive MPP, although these factors may depend on sampling time and local epidemiology (17–20).

Several retained predictors are clinically plausible but nonspecific. ALT, AST, albumin, and BUN may reflect systemic involvement, hepatic stress, nutritional or inflammatory status, and overall illness burden. White blood cell and platelet counts may capture inflammatory or hematologic responses, whereas cough duration and non-definitional respiratory findings such as wheezing and lung rales may reflect clinical course without directly duplicating the outcome definition. These associations should be interpreted as supportive signals within a multivariable model, not as independent diagnostic criteria for SMPP (21–23).

### Comparison with conventional clinical assessment and machine-learning approaches

Conventional clinical recognition of SMPP depends on the physician's synthesis of fever course, respiratory status, hypoxemia, inflammatory markers, imaging findings, complications, and treatment response. The revised model does not replace this process. Rather, by applying a strict non-overlap feature set, it provides a more conservative estimate of whether routinely available non-definitional variables can contribute to standardized risk estimation. This approach reduces the possibility that model performance is driven only by variables already used to define the outcome. Recent studies have also used machine learning to predict multilobar pulmonary consolidation and to construct models based on routine blood parameters, supporting the value of structured multivariable analysis in pediatric MPP (24, 25).

Another important revision is the explicit removal of leakage and outcome-overlapping variables. Data leakage in clinical machine-learning studies can occur when model-derived risk scores, post-diagnostic labels, predicted probabilities, or variables unavailable at the intended decision point are inadvertently included during training. Circular reasoning can also occur when variables used to define the outcome are then used as predictors of that same outcome. In the revised analysis, both types of problematic variables were excluded before dataset splitting and model fitting. The resulting performance estimates are therefore more methodologically transparent and less vulnerable to overoptimistic interpretation (26, 27).

### Clinical implications

In clinical settings, the revised model should be viewed as an adjunctive decision-support framework after routine evaluation rather than as an independent diagnostic tool. Children with high estimated probability of SMPP may warrant closer review, reassessment of respiratory status, repeated laboratory evaluation, imaging review, or specialist consultation, depending on the full clinical context. Conversely, a low predicted probability should not override clinical concern in children with rapidly worsening respiratory status, atypical presentations, underlying disease, mixed infections, or other high-risk features. External validation is required before integration into electronic medical record systems (28, 29).

It is recommended, however, that the model be utilized not as a diagnostic tool in and of itself but rather as an addition. In order to properly evaluate a probability output, it is necessary to take into consideration clinical examination, local epidemiology, treatment response, and the judgment of the physician. Children who have atypical presentations, underlying disorders, mixed infections, or a quickly changing respiratory status may require customized assessment even when the model likelihood is not high. This is especially true for children who have complex respiratory conditions. For the purpose of clinical implementation, a low threshold may be set when the objective is to reduce the number of severe cases that are overlooked, whereas a higher threshold may be used when the objective is to prevent excessive triage and the utilization of resources that are not essential.

### Strengths and limitations

This study has several strengths. First, it included 1,046 children and 205 SMPP cases over an 11-year period. Second, multiple machine-learning algorithms were compared rather than relying on a single model. Third, model evaluation included discrimination, calibration, decision curve analysis, threshold-specific performance, and cross-validation. Fourth, the revised analysis directly addressed data leakage and circular reasoning by removing model-derived variables and severity-definition-overlapping predictors. Fifth, an exploratory time-period analysis was added to examine whether performance was broadly stable across pre-COVID-19, pandemic, and post-pandemic periods.

Several limitations should be acknowledged. First, this was a retrospective single-center study, and unmeasured confounding, incomplete documentation, and local practice patterns could not be fully eliminated. Second, the outcome depended on clinician-confirmed severity classification documented over an 11-year span, which may introduce institutional and temporal bias despite the use of guideline-based criteria. Third, although the strict non-overlap analysis reduced circular reasoning, some retained variables may still indirectly reflect illness severity. Fourth, model performance was validated internally only and was not tested in an independent multicenter cohort. Fifth, time-period subgroup analyses were exploratory because each subgroup was relatively small and still derived from the same institution. Sixth, treatment timing, macrolide resistance, dynamic biomarker trajectories, and external radiology-review consistency were not fully incorporated (30). Finally, radiological severity scoring was reconstructed retrospectively from formal reports and available imaging descriptions rather than through a prospective blinded central imaging review. Although we used a prespecified 1–6 scoring rule and a dual-investigator consensus procedure, residual variability in report detail and reader interpretation may remain.

Future research should validate this model in multicenter prospective cohorts and determine whether model-assisted risk stratification improves clinical workflow, reduces delayed recognition of severe disease, or optimizes the use of imaging and laboratory testing. Future studies should also evaluate dynamic prediction models that incorporate changes in clinical status and laboratory markers over time, while carefully defining the intended prediction time point to avoid leakage. Clinical implementation should be supported by calibration monitoring, decision-threshold evaluation, fairness assessment across age groups and clinical settings, and clear guidance that the model is an adjunct to—not a replacement for—clinical judgment.

## Conclusion

In conclusion, after excluding data-leakage variables and severity-definition-overlapping predictors, machine-learning models maintained good internal diagnostic performance for classifying SMPP among children with MPP. Support vector machine achieved the highest AUC in the strict non-overlap test set, while the random forest model provided balanced threshold performance and interpretable feature-importance output. The revised model should be interpreted as an internally validated adjunctive risk-stratification framework rather than a standalone early diagnostic tool. Multicenter external validation and prospective clinical evaluation are necessary before routine use.

## Data Availability

The raw data supporting the conclusions of this article will be made available by the authors, without undue reservation.
