# Supplementary material for: Explainable machine learning for diagnosing severe Mycoplasma pneumoniae pneumonia in children: model development and internal validation
Source: Front Pediatr. 2026 Aug 14;14:1895967. doi: 10.3389/fped.2026.1895967 (PMC13522111; doi:10.3389/fped.2026.1895967)
Supplement: Supplementary file 1 [file Table1.docx]

**Supplementary Table S1. Variable dictionary and missingness after leakage exclusion**

| **Variable** | **Type** | **Coding or unit** | **Missing rate, %** | **Preprocessing in corrected analysis** |
| --- | --- | --- | --- | --- |
| Age | Continuous | Months | 0.0 | Included |
| Sex | Binary | Male=1, Female=0 | 0.0 | One-hot/binary coding |
| Fever duration | Continuous | Days | 2.5 | Multiple imputation |
| Cough duration | Continuous | Days | 3.3 | Multiple imputation |
| Maximum temperature | Continuous | °C | 1.9 | Multiple imputation |
| Wheezing | Binary | Yes=1, No=0 | 1.7 | Mode imputation |
| Dyspnea | Binary | Yes=1, No=0 | 1.5 | Mode imputation |
| Poor mental status | Binary | Yes=1, No=0 | 1.6 | Mode imputation |
| Lung rales | Binary | Yes=1, No=0 | 2.4 | Mode imputation |
| SpO2 | Continuous | % | 4.8 | Multiple imputation |
| Imaging score | Ordinal | 1–6 points | 3.6 | Multiple imputation |
| Pleural effusion | Binary | Yes=1, No=0 | 2.1 | Mode imputation |
| Extrapulmonary complications | Binary | Yes=1, No=0 | 1.8 | Mode imputation |
| Coinfection | Binary | Yes=1, No=0 | 5.4 | Mode imputation |
| WBC | Continuous | ×10⁹/L | 3.1 | Multiple imputation |
| Neutrophil percentage | Continuous | % | 3.9 | Multiple imputation |
| Platelet | Continuous | ×10⁹/L | 4.7 | Multiple imputation |
| CRP | Continuous | mg/L | 4.2 | Multiple imputation |
| SAA | Continuous | mg/L | 7.9 | Multiple imputation |
| LDH | Continuous | IU/L | 4.5 | Multiple imputation |
| ALT | Continuous | U/L | 5.7 | Multiple imputation |
| AST | Continuous | U/L | 5.9 | Multiple imputation |
| BUN | Continuous | mmol/L | 6.6 | Multiple imputation |
| Albumin | Continuous | g/L | 5.2 | Multiple imputation |
| D-dimer | Continuous | mg/L | 8.1 | Multiple imputation |
| IL-6 | Continuous | pg/mL | 10.8 | Multiple imputation |
| Risk_score_sim and model-prediction columns | Derived/leakage | Not clinical baseline variables | 0.0 | Excluded from corrected model |

Note: The corrected analysis excluded Risk_score_sim, *_pred_probability, *_predicted_label, and Prediction_note columns to avoid label leakage. Values are presented as mean ± SD, median (IQR), or n (%), as appropriate.
