# Supplementary material for: Explainable machine learning for diagnosing severe Mycoplasma pneumoniae pneumonia in children: model development and internal validation
Source: Front Pediatr. 2026 Aug 14;14:1895967. doi: 10.3389/fped.2026.1895967 (PMC13522111; doi:10.3389/fped.2026.1895967)
Supplement: Supplementary file 2 [file Table2.docx]

**Supplementary Table S2. Machine-learning algorithms and main hyperparameters**

| **Model** | **Main hyperparameters used in corrected analysis** |
| --- | --- |
| Logistic regression | L2 penalty; class_weight=balanced; max_iter=1000 |
| Random forest | n_estimators=500; max_depth=6; min_samples_leaf=10; class_weight=balanced |
| XGBoost | n_estimators=300; learning_rate=0.03; max_depth=3; subsample=0.8; colsample_bytree=0.8 |
| LightGBM | n_estimators=300; learning_rate=0.03; max_depth=4; num_leaves=15; class_weight=balanced |
| Support vector machine | RBF kernel; C=1.0; probability=True; class_weight=balanced |
| k-nearest neighbors | n_neighbors=15; weights=distance |
| Naive Bayes | GaussianNB |
| Decision tree | max_depth=4; min_samples_leaf=20; class_weight=balanced |

Note: The corrected analysis excluded Risk_score_sim, *_pred_probability, *_predicted_label, and Prediction_note columns to avoid label leakage. Values are presented as mean ± SD, median (IQR), or n (%), as appropriate.
