# Supplementary material for: Explainable machine learning for diagnosing severe Mycoplasma pneumoniae pneumonia in children: model development and internal validation
Source: Front Pediatr. 2026 Aug 14;14:1895967. doi: 10.3389/fped.2026.1895967 (PMC13522111; doi:10.3389/fped.2026.1895967)
Supplement: Supplementary file 3 [file Table3.docx]

**Supplementary Table S3. Five-fold cross-validation AUC in the training set for the strict non-overlap analysis**

| **Model** | **Mean CV AUC** | **SD** |
| --- | --- | --- |
| Random forest | 0.944 | 0.025 |
| XGBoost | 0.936 | 0.028 |
| LightGBM | 0.938 | 0.022 |
| Logistic regression | 0.949 | 0.029 |
| Support vector machine | 0.951 | 0.027 |
| K-nearest neighbors | 0.908 | 0.030 |
| Decision tree | 0.864 | 0.023 |
| Naive Bayes | 0.932 | 0.034 |

Note: Cross-validation was performed only within the training set after all exclusion rules for the strict non-overlap analysis were defined.
