# Supplementary material for: Explainable machine learning for diagnosing severe Mycoplasma pneumoniae pneumonia in children: model development and internal validation
Source: Front Pediatr. 2026 Aug 14;14:1895967. doi: 10.3389/fped.2026.1895967 (PMC13522111; doi:10.3389/fped.2026.1895967)
Supplement: Supplementary file 4 [file Table4.docx]

**Supplementary Table S4. Confusion matrices and diagnostic performance in the test set**

| **Model** | **TN** | **FP** | **FN** | **TP** | **Accuracy** | **Sensitivity** | **Specificity** |
| --- | --- | --- | --- | --- | --- | --- | --- |
| Random forest | 221 | 32 | 12 | 49 | 0.860 | 0.803 | 0.874 |
| XGBoost | 220 | 33 | 13 | 48 | 0.854 | 0.787 | 0.870 |
| LightGBM | 220 | 33 | 14 | 47 | 0.850 | 0.770 | 0.870 |
| Support vector machine | 216 | 37 | 17 | 44 | 0.828 | 0.721 | 0.854 |
| Logistic regression | 213 | 40 | 18 | 43 | 0.815 | 0.705 | 0.842 |
| Naive Bayes | 210 | 43 | 16 | 45 | 0.812 | 0.738 | 0.830 |
| k-nearest neighbors | 212 | 41 | 20 | 41 | 0.806 | 0.672 | 0.838 |
| Decision tree | 206 | 47 | 19 | 42 | 0.790 | 0.689 | 0.814 |

Note: The corrected analysis excluded Risk_score_sim, *_pred_probability, *_predicted_label, and Prediction_note columns to avoid label leakage. Values are presented as mean ± SD, median (IQR), or n (%), as appropriate.
