# Supplementary material for: Explainable machine learning for diagnosing severe Mycoplasma pneumoniae pneumonia in children: model development and internal validation
Source: Front Pediatr. 2026 Aug 14;14:1895967. doi: 10.3389/fped.2026.1895967 (PMC13522111; doi:10.3389/fped.2026.1895967)
Supplement: Supplementary file 5 [file Table5.docx]

**Supplementary Table S5. Time-period performance of the strict non-overlap random forest model in the internal test set**

| **Time period** | **N** | **SMPP** | **AUC** | **Sensitivity** | **Specificity** | **Accuracy** | **TN/FP/FN/TP** |
| --- | --- | --- | --- | --- | --- | --- | --- |
| 2015-2019 | 130 | 29 | 0.911 | 0.862 | 0.901 | 0.892 | 91/10/4/25 |
| 2020-2022 | 63 | 13 | 0.951 | 0.846 | 0.920 | 0.905 | 46/4/2/11 |
| 2023-2026 | 69 | 9 | 0.944 | 0.667 | 0.933 | 0.899 | 56/4/3/6 |

Note: This additional analysis was prepared to address Reviewer 2's concern that the study period spans pre-COVID-19, pandemic, and post-pandemic eras. The estimates are exploratory because each time-period subgroup is relatively small and still comes from the same single-center internal test set.
